# Predicting normative walking biomechanics across the lifespan using seven simple features

**DOI:** 10.1186/s12984-025-01768-9

**Published:** 2025-10-24

**Authors:** Bernard X. W. Liew, Rachel Senden, David Rugamer, Emanuel Sommer, Kenneth Meijer, Qichang Mei, Richard Foster, Matthew Taylor

**Affiliations:** 1 School of Sport, Rehabilitation and Exercise Sciences, University of Essex, Colchester, Essex UK; 2 Department of Physical Therapy, Maastricht University Medical Center, Maastricht, Limburg The Netherlands; 3 Department of Statistics, Ludwig-Maximilians-Universität München, Munich, Germany; 4 Department of Nutrition and Movement Sciences, NUTRIM Research Institute of Nutrition and Translational Research in Metabolism, Maastricht University Medical Centre+, Maastricht, The Netherlands; 5 Faculty of Sports Science, Ningbo University, Ningbo, China; 6 Auckland Bioengineering Institute, The University of Auckland, Auckland, New Zealand; 7 Research Institute for Sport and Exercise Sciences, Liverpool John Moores University, Liverpool, UK

**Keywords:** Functional regression, Kinematics, Kinetics, Lifespan, Walking speed

## Abstract

The assessment of gait impairments requires a normative reference for comparison. For a fair assessment, comparisons must be made against a reference standing after controlling for sex, anthropometry, and walking characteristics. This study aimed to develop statistical models that predict the lower-limb kinematics and kinetics of walking across the lifespan of healthy participants, using seven simple covariates. Sixteen statistical models predicted 16 joint kinematics and kinetics during walking using the covariates of sex, age, height, mass, side (laterality), walking speed, and cadence, which were developed based on 301 participants between three to 91 years old. The root mean squared error (RMSE) ranged from 4.71° to 7.97° for joint angles, within 0.07 N/kg for ground reaction forces, 0.09 to 0.15 Nm/kg for joint moments, and 0.33 to 0.39 W/kg for joint powers. We provide both online and local apps which can be easily used by clinicians and scientists to generate normative walking data with uncertainty values, which can be used for movement impairment analysis (https://github.com/EmanuelSommer/ShinyFOSR) .

## Introduction

Movement impairments are common in many neurological [1], musculoskeletal [2], cardiovascular [3] disorders, and even manifest naturally due to ageing [4]. The traditional approach to assessing movements occurs in a lab, involves three-dimensional motion capture using multiple optical cameras and force plates [5]. With rapid advancements in computer vision and sensor technologies, movement assessments can now be easily undertaken in clinical environments, using ubiquitous technologies, requiring no specialised training [6]. Assessing movement impairments in patients requires the comparison with typical reference standards from a healthy person or cohort [7, 8]. To ensure a correct interpretation, comparisons must be made with healthy controls after controlling for sex, anthropometry (e.g. body mass index, height), and spatiotemporal walking characteristics (e.g. walking speed) [9]. To our knowledge, there are very few tools that non-specialists can leverage to provide that baseline reference to make clinical judgements.

The most common method to compare movement impairments is based on tools, termed “gait indices”, such as the Gait Deviation Index [10–12], Gait Profile Score [13], and the Gillette Gait Index [10]. A challenge when using traditional gait indices is that it requires clinicians or researchers to collect data on individuals defined as “normative”, from scratch, to develop a database upon which a normative index is quantified and compared. This process can be very time-consuming and expensive. To our knowledge, no open-source “normative” dataset or normative gait indices have been published to enable researchers to compare. Another limitation of traditional gait indices is that typical movements can be affected by movement-specific factors, like the speed of walking [14], and potentially individual-specific factors, like age. This means that if the gait index was developed based on a sample with a different characteristic from the target patient, or performed a movement with different task requirements, traditional gait indices may classify the target patient as impaired.

An alternative to the use of traditional gait indices is a direct population-based statistical approach towards quantifying an average with a measure of uncertainty of any biomechanical parameters of interest [9, 15, 16]. Population-based statistical approaches in biomechanics, such as Statistical Shape Modelling, are not new and have been largely used to scale the morphology of differential anatomical region(s) from a generic model to fit the anthropometry of the measured individual [17]. Less research has focused on the statistical prediction of normal human motion, particularly via predictive factors that are simple, quick, and cheap to measure clinically. A previous study reported that the sagittal plane joint angles during walking of the ankle, knee, and hip could be predicted within < 2°, based only on knowing an individual’s walking speed, gender, age, and body mass index [9]. Another study reported that lower-limb joint angles can be predicted within a root-mean-squared error (RMSE) of 4.45° to 6.61° based on age, height, and weight, or based on the shape of the lower-limb bones [18].

A limitation of prior studies [9, 18] is the inclusion of a restricted age spectrum of participants (e.g. 39 ± 13 years in [9], and 22 ± 2 years in [18]), the assumption of statistical linearity between the covariates (also known as predictors) and the biomechanical outcomes and the inclusion only of kinematic features as outcomes for prediction. It is well known that kinematic and kinetic gait differences can be observed across the human lifespan [19]. For example, in a study, only children reported that ankle push-off power (A2) appeared to mature by 4 years old [20]. Separate research only on adults (i.e. ≥ 18 years old) reported that A2 power peaked again at 60 years [19]. A population-based statistical model of gait biomechanics must include participants from a wider age spectrum of young children to very old adults. Previous studies also assumed a linear relationship between the included covariates and biomechanical outcomes using statistical techniques like partial least squares regression [18] and linear stepwise regression [9]. However, previous studies have reported nonlinear relationships between covariates such as age and biomechanical features like joint power [19]. In other words, the association between a covariate and a predictor varies depending on the value of the covariates.

This study aims to develop statistical models that non-specialists can use to generate normative kinematic and kinetic data during walking, to (1) provide a baseline reference for comparison, and (2) use it as a tool for clinical and academic education. We will do so by creating models that predict the lower-limb kinematics and kinetics of walking across the lifespan of healthy participants, using simple covariates that can be easily measured in clinical settings, such as sex, age, height, mass, side (laterality), walking speed, and cadence. We hypothesised that the root-mean-squared error (RMSE) for the predicted lower-limb joint angles would be less than the previously defined minimal detectable change threshold of ≤ 5° [21]. For joint kinetics, we also hypothesise that the RMSE of the predicted joint moments would be less ≤ 0.4 Nm/Kg [21].

## Methods

### Participants

Normative data collected from previous studies of Senden et al. were used for this study, and the two studies included 55 typically developed children [22] and 246 healthy adults [23].

### Data collection

Three-dimensional (3D) gait analysis was performed at the motion lab of the MUMC + using the Computer Assisted Rehabilitation Environment (CAREN, Motek Medical BV, Amsterdam) system. Participants walked on an instrumented split-belt treadmill (ForceLink, Culemborg, 1000 Hz), while marker trajectories were captured with a 12-camera optical motion capture system (Vicon, Oxford, 100 Hz). Participants wore standardised gymnastic shoes and a safety harness to prevent falls.

Reflective markers were placed on the following landmarks according to the Human Body Lower Limb Model (HBM-II) [24]: Xyphoid process, sternum, C7, T10, bilateral anterior and posterior superior iliac spines, and for both legs on the thigh, medial and lateral femoral condyles and malleoli, anterior of the tibia, second toe, 5th metatarsal, and mid-calcaneus [24]. Markers on medial femoral condyles and malleoli were used for calibration only. The pelvis had 6 degrees of freedom (DOF) (3 global translations of pelvis origin and rotation about Z (yaw), Y (pitch), X (roll)), the hip had three rotational DOFs (sagittal, frontal, rotation), the knee had one sagittal DOF (sagittal), and the ankle had two DOF (sagittal and frontal) [24]. The position and orientation of the segments were resolved using an inverse kinematics algorithm [24].

After a six-minute familiarisation period at comfortable walking speed, participants walked for 250 steps at a comfortable, slow (30% slower than comfortable), and fast (30% faster than comfortable) speed, which were performed in a random order. To determine comfortable walking speed, children performed repeated overground walking trials over a nine-meter walkway, while speed was measured using two movement detection ports. For the healthy adults, the RAMP protocol was used where participants started to walk on the treadmill at 0.5 m/s while the speed was gradually increased to 0.01 m/s every second until a comfortable speed was reached. This was repeated three times and the average of three repetitions was used as the comfortable speed.

The force plate configurations for the analog data were set at 10 Hz for the low-pass prefilter frequency and 20 N for the force threshold. Marker trajectories and force plate data were filtered with a unidirectional 2nd order Butterworth filter at 6 Hz. Gait event detection was defined based on a combination of heel marker kinematics and force plate data (exceeding the threshold of 50 N) [25]. Custom Matlab scripts were used to check data quality and calculate spatiotemporal parameters, kinematics, and kinetics. Kinematic and kinetic data were time-normalised to 100 data points between two consecutive initial contacts for each side. GRF, joint moment and power data were subsequently normalised to body mass (kg). The kinematic and kinetic trajectories have been uploaded and are freely available in the Open Science Framework (OSF) (children: https://osf.io/3xqew/; adults: https://osf.io/t72cw/).

### Statistical analysis

All analyses were conducted in R software (v4.4.2) using the *refund* package (v0.1-37). We developed 16 statistical models to predict 16 kinematic and kinetic trajectories – angles (hip flexion, abduction, rotation; knee flexion; ankle plantarflexion and inversion), GRF (vertical and anterior-posterior), moments (hip flexion, abduction, rotation; knee flexion; ankle plantarflexion), and powers (hip, knee, ankle flexion). For all models, the same covariates were included: sex (male or female), age (years), walking speed (m/s), body mass (kg), height (m), cadence (steps/min), side (left or right), and the random effect of the subject. We use a function-on-scalar model for each of the three joints – specifically the *pffr* function in the *refund* package. As a functional additive model, we model the expectation of each outcome trajectory yi(t) for every subject i at gait point t.

$$\begin{aligned} y_{i} \left( t \right) & = \beta _{0} \left( t \right) + {\text{ }}f_{{age}} \left( {age_{i} ,t} \right) + {\text{ }}f_{{speed}} \left( {speed_{i} ,t} \right) \\ & + {\text{ }}f_{{cadence}} \left( {cadence_{i} ,t} \right) + {\text{ }}f_{{ht}} \left( {height_{i} ,t} \right) \\ & + {\text{ }}f_{{mass}} \left( {mass_{i} ,{\text{ }}t} \right) + sex_{i} \beta _{{sex}} \\ & + {\text{ }}side_{i} \beta _{{side}} + {\text{ }}b_{i} \left( t \right) + \varepsilon _{{ij}} \\ \end{aligned} $$

where β_0_(*t*) is the model’s time-varying intercept, β_sex_ a time-independent effect of sex, β_side_ a time-independent effect of side, f(, *t*) indicate functional effects that are estimated to be non-linear both in the direction of the covariate and across time, bi(*t*) are time-varying random intercepts, and ε_i_ and independent Gaussian error term.

An 80:20 split of the data was performed to create a training set (80%, *n* = 240 participants) to develop the models, and a testing set (*n* = 61 participants) to validate the accuracy of the outcome predictions. The prediction performance of the models was determined by comparing the 16 predicted outcomes in the test set, against their original values using the Root Integrated Mean Squared Error (BW), relative Root Integrated Mean Squared Error (relRMSE, %) [26], and Pearson correlation coefficient (cor) [27, 28].

1 $$\:RMSE=\:\sqrt{\frac{{\int\:}_{0}^{T}{[{u}_{obs}\left(t\right)-\:{u}_{pred}\left(t\right)]}^{2}dt}{T}}$$

2 $$\begin{aligned} relRMSE & = ~\frac{{RMSE}}{{0.5\left[ {\mathop \sum \nolimits_{{i = 1}}^{2} \left( {\max _{{0 < t < T}} \left( {u_{i} \left( t \right)} \right) - ~\min _{{0 < t < T}} \left( {u_{i} \left( t \right)} \right)} \right)} \right]}}~ \\ & \times ~100\% \\ \end{aligned}$$

where $\:T$ represents the stance duration between initial contact and toe-off, $\:{u}_{obs}\left(t\right)$ represents the value at the $\:{t}^{th}$time point of the observed outcome, $\:{u}_{pred}\left(t\right)$ represents the value at the $\:{t}^{th}$time point of the predicted outcome, and $\:i$ represents either the observed or predicted outcomes. We also report the association (point estimate and 95% confidence interval [CI]) between the outcomes and each of the fixed effect covariates. For the continuous covariates of age, speed, cadence, height and mass, we selected the 25th, 50th, and 75th quantiles of the values to plot the smooth association estimate.

To make the model open source and easily accessible, we retrained the models using the entire dataset (*n* = 301) and created a shiny app (https://github.com/EmanuelSommer/ShinyFOSR) for readers to input different covariate values to predict the mean waveform value of different kinematic and kinetic outcomes. Readers can use the app to predict, plot, and export the values as a figure and table. We also included a reference with a detailed and easy set of instructions to download and use the app locally on their computer. This local version can predict the mean values and the 95%CI or the standard deviation.

## Results

**Fig. 1:**
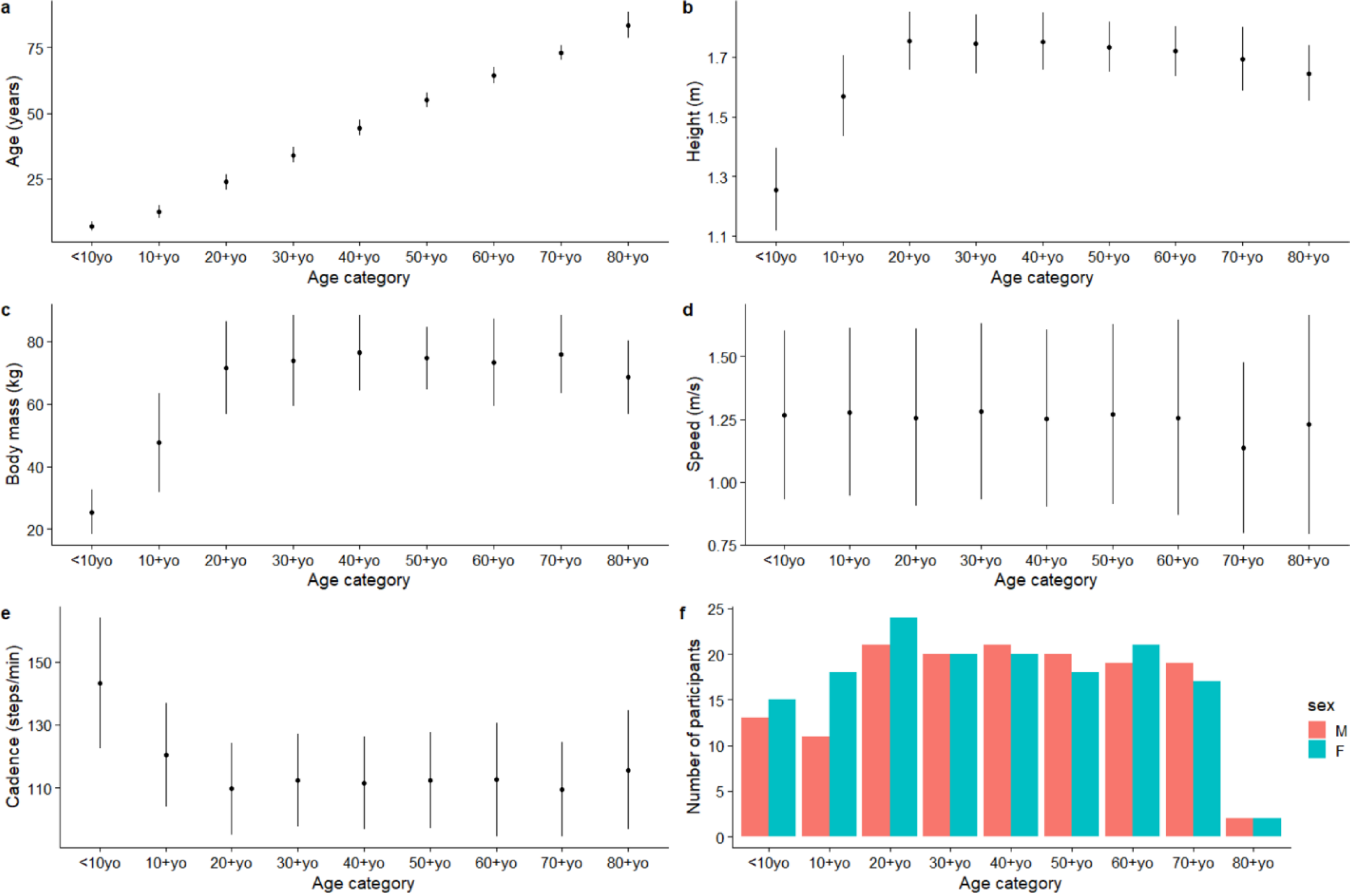
Descriptive characteristics of the included participants (*n* = 301). Point estimate reflect means with error bars representing one standard deviation (a – e), apart from the number of participants (f). Red (male), green (female)

**Fig. 2:**
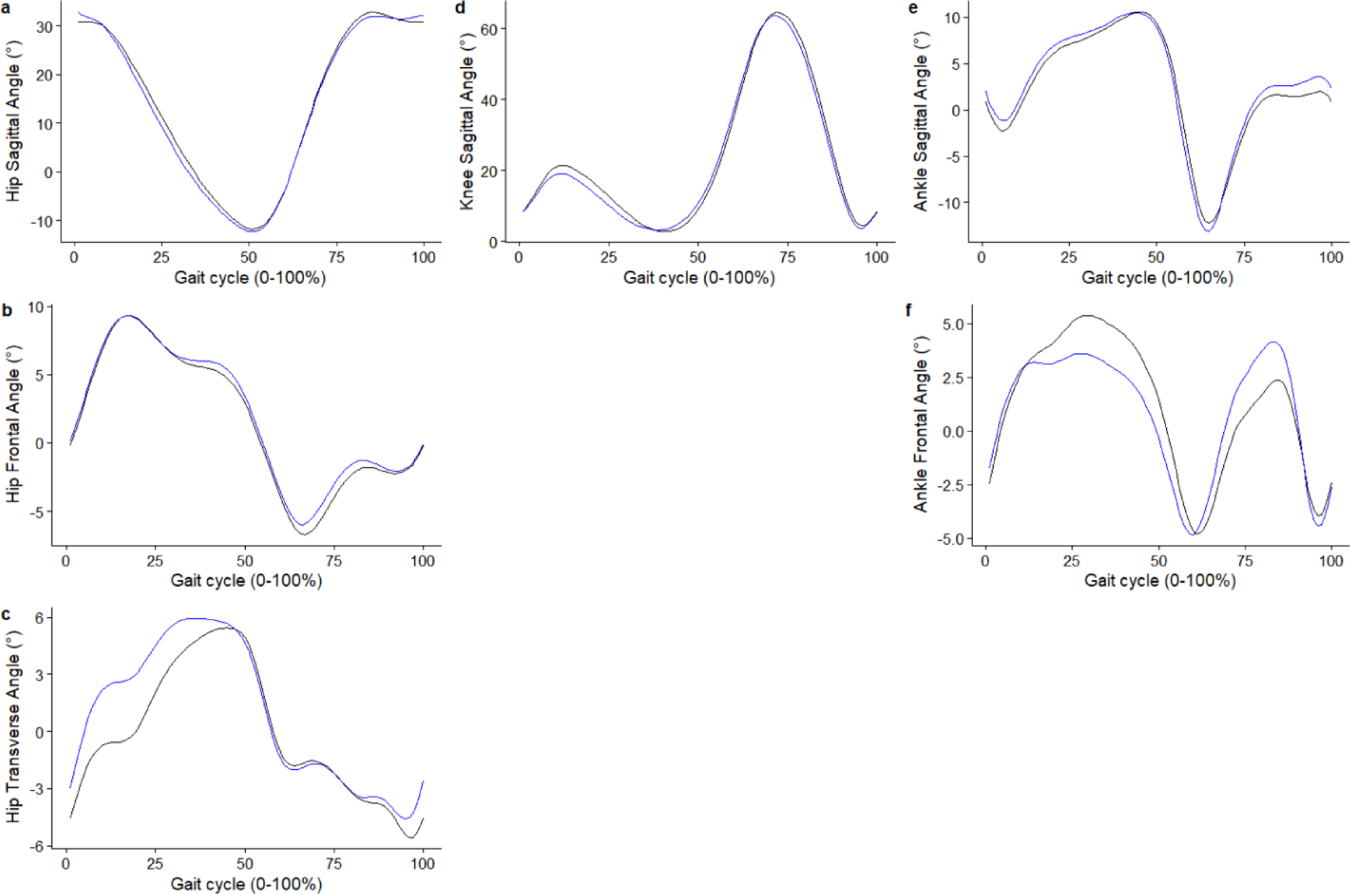
Average predicted (blue) kinematic trajectories against the observed (black) trajectories for the test dataset

**Fig. 3:**
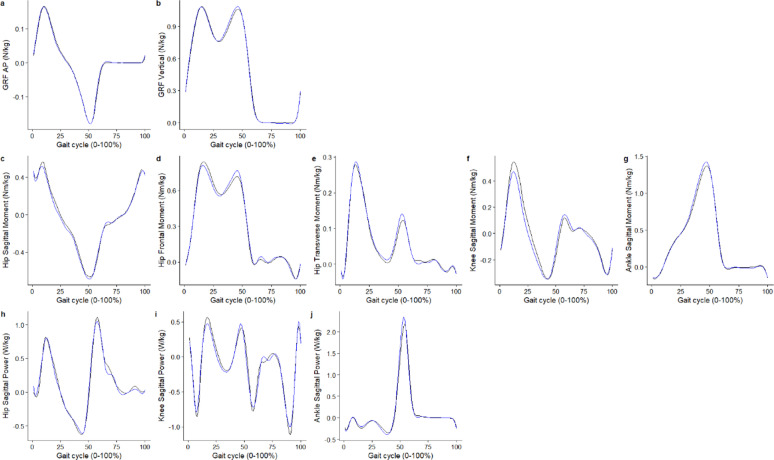
Average predicted (blue) kinetic trajectories against the observed (black) trajectories for the test dataset

Basic descriptive summaries of the cohort can be found in Figure. 1. Figures 2 and 3 illustrate the average predicted kinematic and kinetic trajectories, alongside the observed trajectories in the test dataset. RMSE ranged from 4.71° to 7.97° for kinematic data, while relRMSE ranged from 9.85% to 50.72% (Table 1). Accuracy was the best generally for sagittal plane joint angles and the poorest for transverse plane angles (Table 1). For kinetic data, the statistical models predicted GRFs to be within 0.07 N/kg and < 7% for RMSE, and relRMSE (Table 1). The statistical models were better at predicting joint moments than joint powers, with an accuracy for the former ranging from 0.09 to 0.15 Nm/kg for RMSE and 9.23% to 24.71% for relRMSE; and for the latter ranging from 0.33 to 0.39 W/kg for RMSE and 12.98 to 17.91% for relRMSE (Table 1).

**Table 1:** Accuracy of predicted kinematic and kinetic trajectories

Variables	RMSE	relRMSE (%)	Correlation
Hip Sagittal Angle (°)	7.97 (3.48)	16.59 (8.45)	0.94 (0.06)
Hip Frontal Angle (°)	3.44 (1.45)	21.38 (9.7)	0.89 (0.09)
Hip Transverse Angle (°)	7.94 (3.32)	50.72 (22.1)	0.44 (0.44)
Knee Sagittal Angle (°)	6.21 (2.78)	9.85 (4.7)	0.97 (0.04)
Ankle Sagittal Angle (°)	4.71 (4.13)	18.96 (19.22)	0.88 (0.1)
Ankle Frontal Angle (°)	7.71 (5.85)	45.81 (43.46)	0.38 (0.35)
GRF AP (N/kg)	0.02 (0.01)	6.63 (3.85)	0.96 (0.06)
GRF Vertical (N/kg)	0.07 (0.03)	6.15 (2.7)	0.99 (0.02)
Hip Sagittal Moment (Nm/kg)	0.13 (0.08)	11.07 (7.56)	0.93 (0.11)
Hip Frontal Moment (Nm/kg)	0.12 (0.04)	11.18 (3.8)	0.96 (0.04)
Hip Transverse Moment (Nm/kg)	0.09 (0.03)	24.71 (10.16)	0.79 (0.2)
Knee Sagittal Moment (Nm/kg)	0.15 (0.06)	17.26 (8.38)	0.83 (0.21)
Ankle Sagittal Moment (Nm/kg)	0.15 (0.07)	9.23 (5.13)	0.96 (0.06)
Hip Sagittal Power (W/kg)	0.33 (0.18)	17.61 (7.22)	0.84 (0.15)
Knee Sagittal Power (W/kg)	0.34 (0.15)	17.91 (5.18)	0.78 (0.16)
Ankle Sagittal Power (W/kg)	0.39 (0.18)	12.98 (6.18)	0.79 (0.25)

RMSE – root mean squared error; relRMSE – relative root mean squared error; AP – anterior-posterior; GRF – ground reaction force.

### Smooth effect plots

**Fig. 4:**
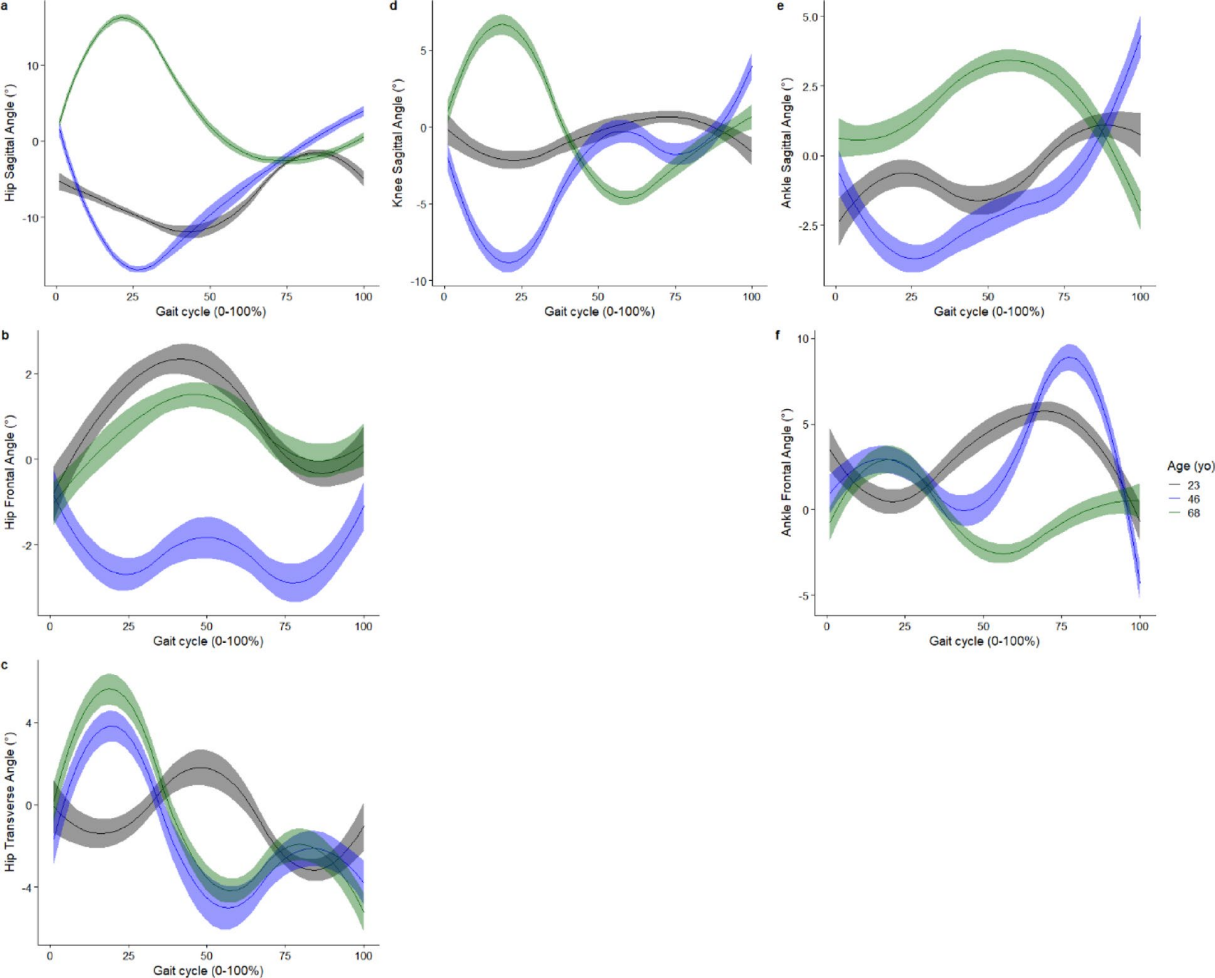
Smooth plots with error clouds as 95% confidence interval for the covariate of age and joint kinematics. Black (23 years old), blue (46 years old), green (68 years old)

**Fig. 5:**
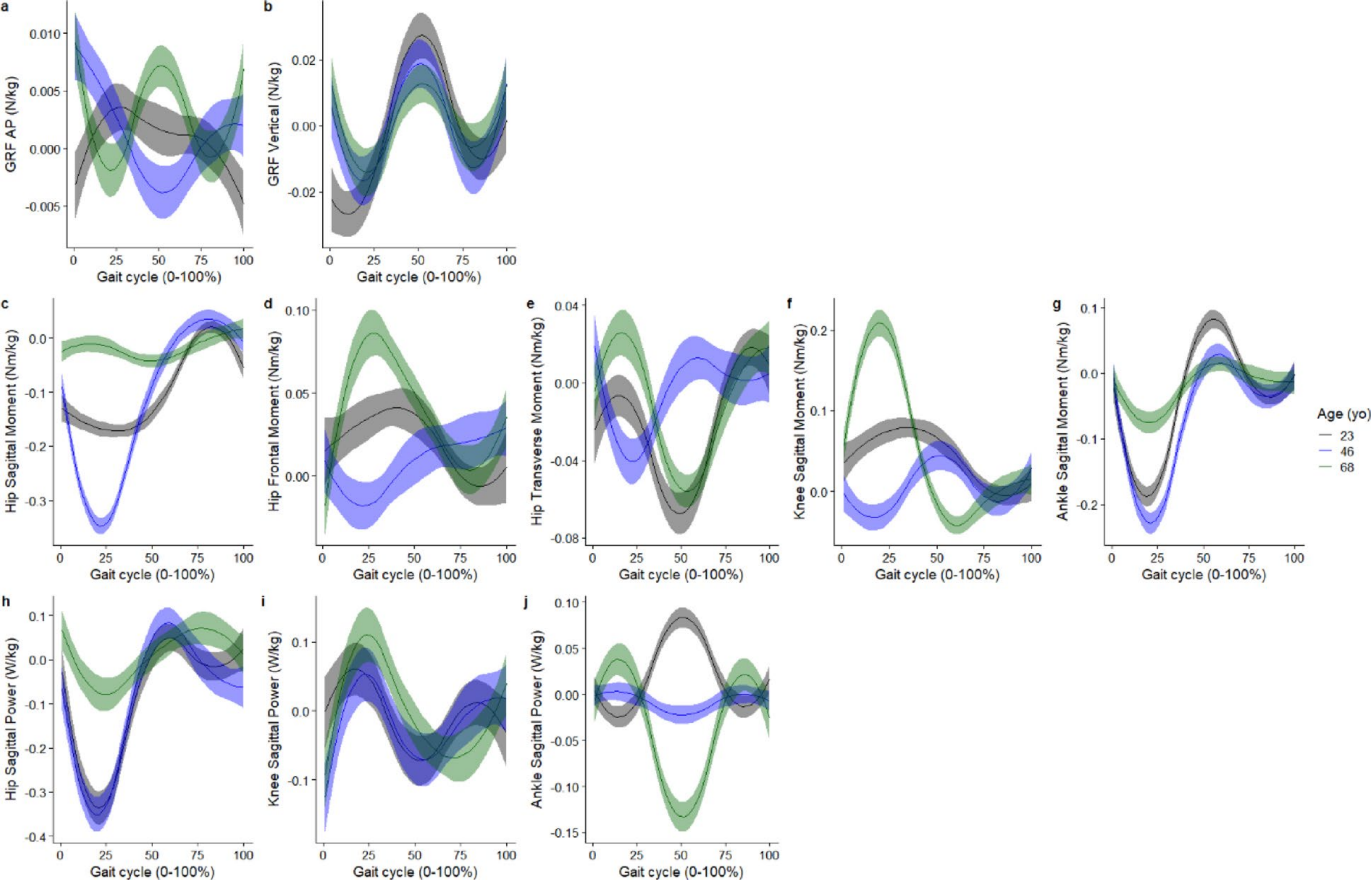
Smooth plots with error clouds as 95% confidence interval for the covariate of age and joint kinetics. Black (23 years old), blue (46 years old), green (68 years old)

The smooth effect plots support the nonlinear association between the included covariates of age and speed and the kinematic and kinetic outcomes. For the covariate age, the greatest effect is on the hip sagittal plane angle at 26% of the gait cycle, where at the age of 46 years it was associated with greater hip extension angle by -16.9° (95%CI -17.3° to -16.5°) (Fig. 4). For joint moments, age had the greatest effect on the hip sagittal moment, where at age 46 years, it was associated with greater hip flexion moment by -0.35Nm/kg (95%CI -0.36 to -0.33Nm/kg) at 21% of the gait cycle (Fig. 5). Age also had the greatest effect on hip flexion power, where at age 46 years, it was associated with greater hip power absorption by -0.35 W/kg (95%CI -0.39 to -0.31 W/kg) at 19% of the gait cycle (Fig. 5).

**Fig. 6:**
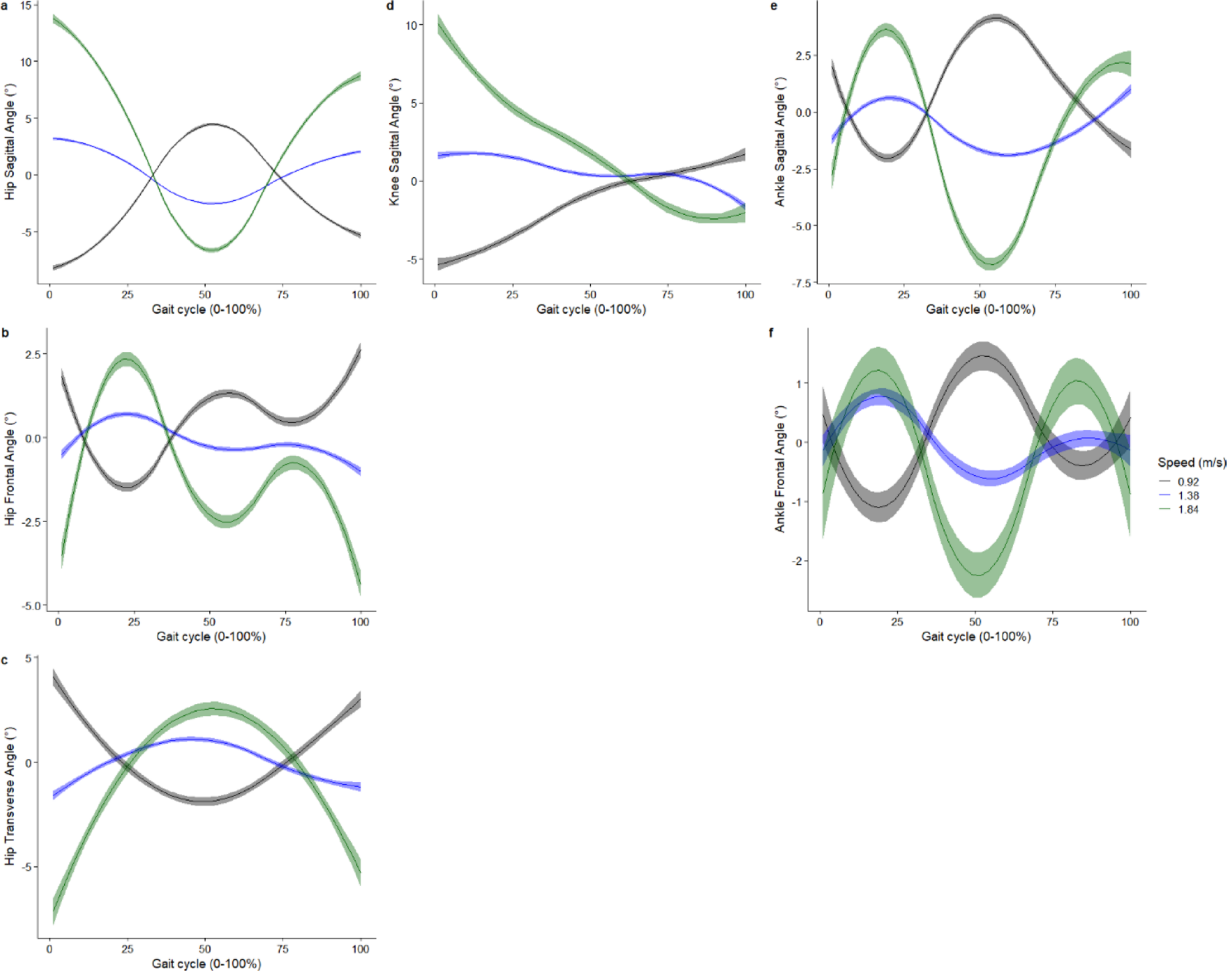
Smooth plots with error clouds as 95% confidence interval for the covariate of speed and joint kinematics. Black (0.92 m/s), blue (1.38 m/s), green (1.64 m/s)

**Fig. 7:**
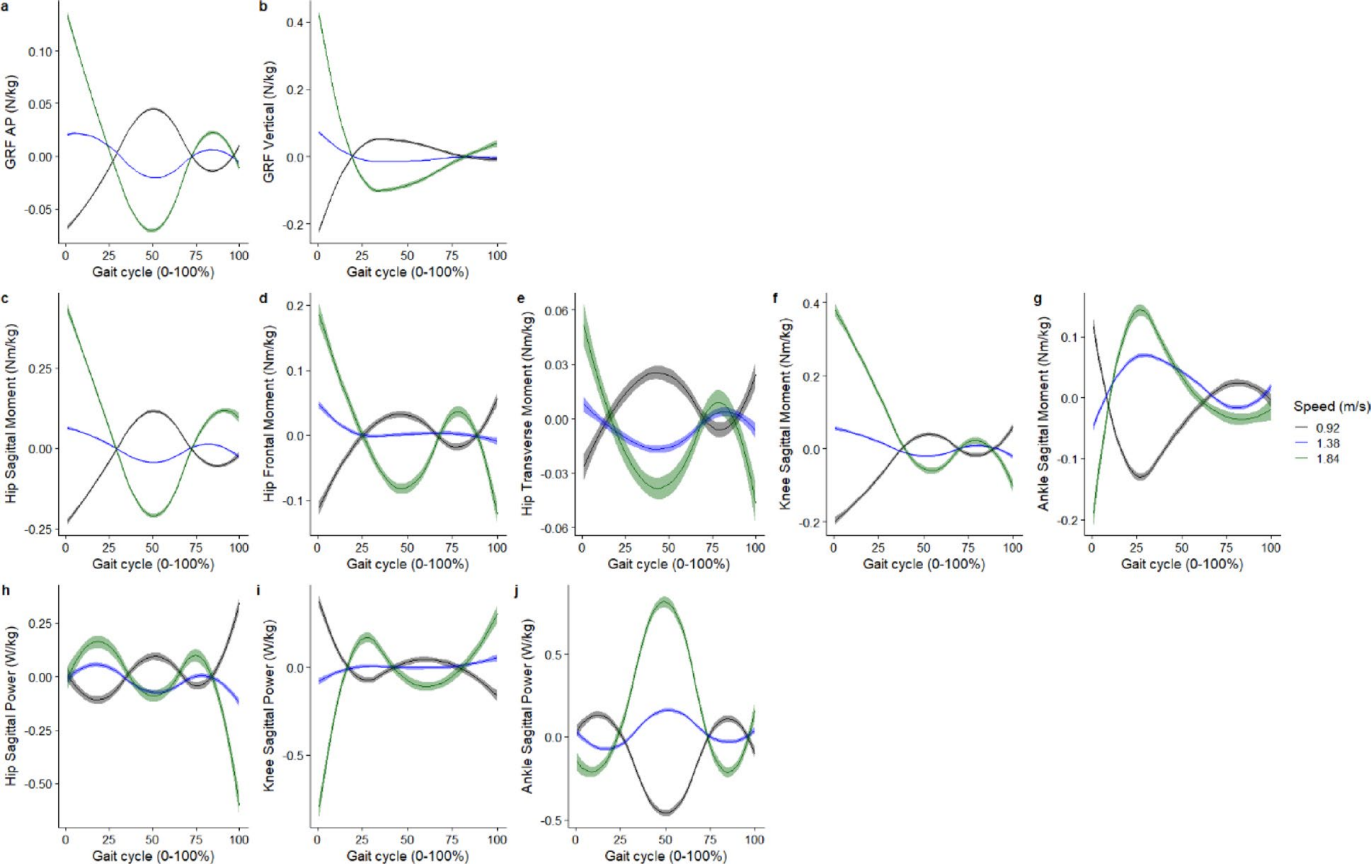
Smooth plots with error clouds as 95% confidence interval for the covariate of speed and joint kinetics. Black (0.92 m/s), blue (1.38 m/s), green (1.64 m/s)

For the covariate speed, the greatest effect is on the hip sagittal angle at 1% of the gait cycle, where with a speed of 1.84 m/s, it was associated with an increase in hip flexion angle 13.4° (95%CI 13.4° to 14.2°) (Fig. 6). For joint moments, speed had the greatest effect on the hip sagittal moment, where at a speed of 1.84 m/s, it was associated with an increase in hip extensor moment by 0.43Nm/kg (95%CI 0.42 to 0.45Nm/kg) at 1% of the gait cycle (Fig. 7). Speed had the greatest effect on ankle plantarflexion power, where at a speed of 1.84 m/s it was associated with an increase in power generation by 0.82 W/kg (95%CI 0.79 to 0.85 W/kg) at 49% of the gait cycle (Fig. 7).

## Discussion

Determination of impaired movement depends on knowing normal movement. Statistical modelling based on simple clinical measures represents a simple way that scientists and clinicians can predict key walking biomechanics of healthy individuals. The RMSE averaged across the stride cycle for the lower-limb joint angles was < 8°, poorer than our hypothesis of < 5°, and also marginally poorer than prior studies < 7° [18]. In support of our hypothesis, the RMSE was between 0.09 and 0.15 Nm/kg for joint moments, and this was also better than prior studies [29].

The predicted RMSE for joint angles in this study was approximately 5° greater than previous studies, which reported that the RMSE for sagittal plane joint angles was within 2° [9, 30]. Previous studies have reported that the minimal detectable change values of lower-limb joint angles during walking of ~ 5°, but can increase up to 7° [21]. RMSE values greater than minimal detectable change values suggest some biomechanical outcomes should be used with caution. For example, readers should have more confidence in using our models to generate normative ankle plantarflexion angles (≤ 5°), and use more caution when using them to create hip axial rotation angles (≥ 5°) (Table 1). However, even though the error metrics presented reflect the waveform-averaged, the accuracy of our prediction models is not uniform across the stride cycle, as observed in Figures. 2 and 3, with better accuracy in some phases of the stride cycle than in other phases. Also, there is no consensus on a single clinically meaningful threshold kinematic and kinetic value, which is likely to vary depending on the clinical condition and joints investigated. Users of the present prediction models in defining normative walking gait biomechanics should be aware of our reported RMSE values and determine clinically if these errors exceed a clinically meaningful threshold.

The differences in the predicted accuracy of our biomechanical waveforms between studies may be due to different validation methods [9, 30]. Previous studies used leave-one-subject-out cross-validation (LOSOCV) as their validation approach [9, 30], whereas the present study used a train-test split approach. LOSOCV uses more data, apart from one subject, for training. This means that a greater percentage of the data was used for training, and a smaller percentage of data was used to validate the model, compared to a train-test split validation approach. For example, the study of Moissenet et al. [9] used 1325 observations (53 subjects, 5 speeds, 5 trials) for each training loop, whereas we used only 720 observations (240 subjects, 3 speeds) for training. The present findings thus provide a more conservative estimate of gait kinematics predictive modelling, which may be more transferable to unseen real-world applications.

The present study demonstrates that normative joint kinetics during walking can be predicted using simple clinical measures, without having to rely on more complex biomechanical inputs as predictors. The predicted RMSE for GRF, moments, and powers in this study ranged between 0.02 and 0.07 N/kg, 0.09 to 0.15Nm/kg, and 0.33 to 0.39 W/kg, respectively. Surprisingly, these error ranges were less than machine learning prediction models of joint moments using 3D motion capture inputs in running (RMSE: 0.07 to 0.54Nm/kg) [29], and less than models using inertial measurement units (IMUs) as inputs for walking (RMSE: 0.07 to 0.24 Nm/kg) [31]. Wouda et al. predicted vertical GRF to a RMSE of < 0.27BW using three IMU inputs for running [32]. Another study predicted ankle joint power for walking using IMU sensors with a RMSE of < 0.21 W/kg [33]. Statistical models like the present and others [9], are useful for population average prediction – which cannot capture variation between movement cycles, whereas more complex prediction models built upon biomechanical input predictors may be more useful for capturing cycle-specific predictions.

Whilst the present study did not specify apriori a linear relation, previous studies have assumed a linear relationship between the predictors investigated (e.g. age, speed) and the biomechanical outcomes [9, 16], whilst another study assumed a quadratic association with the outcomes [30]. The present study reported a non-linear association between age and ankle plantar flexion, given that ankle plantar flexion increased between 23 and 48 years old and decreased from 48 to 68 years old (Fig. 4e). The non-linear association between age and our 16 biomechanical outcomes investigated was evident in our smooth plots (Figs. 4 and 5). Studies which used linear regression methods reported non-significant linear associations between age and ankle plantar flexion during push-off [9], while other studies reported a decreased ankle plantar flexion during push-off in older adults compared to younger adults [34]. This study provides evidence that it may not be appropriate to assume linearity across all values of the covariates and the outcome across a gait cycle. Hence, whilst linear-regression-based approaches provide very interpretable results (e.g. a beta coefficient), they may not adequately model the relationships between the covariates and the biomechanical outcomes.

A strength of the present study is the fact that we included 301 participants between three and 91 years old, which represents a five times greater sample size than recent studies [9]. Apart from the age category of >80 years old, the current study included >20 participants in each decade of years for adults, included in the modelling, making our statistical model more representative of a lifespan population cohort. Non-biomechanical specialists can use our models and online tool to quickly generate data of normal walking joint kinematics and kinetics using simple clinically oriented variables. This is more advantageous than existing metrics like the Gait Deviation Index, which still requires data collection to establish a reference, nor can such metrics be tuned to key personal and gait characteristics easily. For clinicians, the normative data can be used to compare biomechanical values from patients collected either using traditional motion capture or more recent portable technologies, like OpenCap. Movement impairment assessments may be used as an outcome measure, for prognosis, and even for treatment-decision making. Our lifespan approach also means that clinicians can now account for maturation changes when making clinical judgements in children and adolescents. Lastly, clinical and academic educators may use our models for teaching purposes to visualise normal walking biomechanics.

This study has some limitations. First, the included participants were collected whilst walking on a treadmill, which might differ from overground walking [35]. Differences between treadmill and overground walking ranged from 0.84° (pelvic tilt) to 6.42° (peak knee flexion during swing) for joint angles, and 0.02 Nm/kg (peak knee extension moment) to 0.32 Nm/kg (peak hip extension moment) for joint moment [35]. Second, although we included covariates which have previously been shown to have a significant association with walking biomechanics, more variables may play an important role when developing population-based models which were not currently measured, like ethnicity [36] and psychological factors [37]. Third, our biomechanical model had only one DOF and two DOFs at the knee and ankle, respectively. This means that statistical models to predict the constrained knee and ankle kinematics and kinetics were not generated. Fourth, segment kinematics, like that of the pelvis, were not reported, given that the focus of this paper was on joint kinematics and kinetics. Lastly, our biomechanical model was not scaled geometrically to account for factors like tibial torsion, which has a significant effect on lower-limb joint kinematics and kinetics [38, 39]. Also, we did not assess anthropometric measurements like femoral and tibial torsion angles to use within our statistical models. Although this may increase the performance of our models, it would also make them more complex to use clinically.

## Conclusions

Statistical models using seven clinical covariates can predict most normative walking kinematics and kinetics, apart from non-sagittal plane kinematics, to a level comparable to more complex machine learning models using biomechanical covariates. Both our online and local apps can be used by clinicians and scientists to generate normative data with uncertainty values, which can be plotted against patient data for reporting.

## Data Availability

The data is fully open access as reported in the manuscript hyperlinks.
